# Systemic recurrence of endometrial cancer more than 10 years after hysterectomy: a report of two cases and a brief review of the literature

**DOI:** 10.1186/s43046-020-00052-2

**Published:** 2020-11-02

**Authors:** Leonardo Muratori, Paola Sperone, Gabriella Gorzegno, Anna La Salvia, Giorgio Vittorio Scagliotti

**Affiliations:** 1 Medical Oncology, Department of Oncology, Azienda Ospedaliera Universitaria San Luigi Gonzaga, University of Turin, Regione Gonzole 10, 10043 Orbassano, Turin, Italy; 2 Medical Oncology, Department of Oncology, Hospital Universitario 12 de Octubre, Avenida Cordoba, s/n, 28041 Madrid, Spain

**Keywords:** Endometrial, Carcinoma, Cancer, Relapse, Recurrence

## Abstract

**Background:**

Endometrial carcinoma is one of the most common female cancers in developed countries. Disease stage is associated with the risk of disease relapse after radical treatment. Typically, the risk of disease relapse peaks at 3 years from local radical treatment and then diminishes over time, so that late relapses (i.e., from year 5 afterward) are extremely infrequent. Here, we report two cases of women with endometrial cancer who developed a disease relapse more than 15 years after radical treatment. A review of the literature revealed other seven reports of women with relapse from endometrial cancer occurring more than 10 years after radical treatment.

**Case presentation:**

Case report 1 is a 56-year-old woman with an endometrioid cancer who underwent a hysterectomy with bilateral salpingo-oophorectomy in 1998. She relapsed in the lung in 2014, 16 years from radical surgery. Case report 2, a 75-year-old woman, with an endometrioid cancer, was treated by hysterectomy with bilateral salpingo-oophorectomy and adjuvant radiotherapy. The disease relapse in the lung was detected in 2019, 22 years from radical treatment.

**Conclusion:**

Although guidelines do not support oncological follow-up beyond 5 years from surgery, oncologists should consider late recurrence of endometrial carcinoma in the differential diagnosis of women presenting with metastases of uncertain origin and prior history of this disease.

## Background

Endometrial carcinoma represents the sixth most common neoplasm among women worldwide, and the fourth most common in developed countries [1]. Approximately 98,000 new cases of endometrial carcinoma and 23,000 related deaths are registered in Europe every year. The prognosis of this neoplasm is strictly related to the disease stage at diagnosis. According to the International Federation of Gynecology and Obstetrics (FIGO) staging [2], patients with stage I and II cancers have an excellent prognosis after surgery and adjuvant radiotherapy, with a 5-year survival rate of 80-90% and a recurrence rate between 3 and 15% [3, 4]. Long-term survival rapidly decreases below 25% in advanced stages (FIGO III and IV). Almost all disease relapses occur within 5 years from radical treatment, with increased frequency in the first 3 years [5]. Just a few cases of relapse beyond year five are reported in the medical literature. Here, we describe two women with endometrial cancer who developed systemic relapse after 16 and 22 years from radical surgery and provide a brief overview of the medical literature on similar cases.

## Case presentation

We describe the clinical history of two patients with late recurrence of endometrial cancer, treated, and followed at our center. We also performed a systematic PubMed search for case reports of case series of late (beyond year ten from treatment) systemic relapse from endometrial cancer. We focused only on recurrences from endometrial cancer occurring after radical surgery and excluded new primary endometrial cancers developing in extrauterine endometriosis many years after a hysterectomy performed for endometriosis.

### Case report 1

**Fig. 1:**
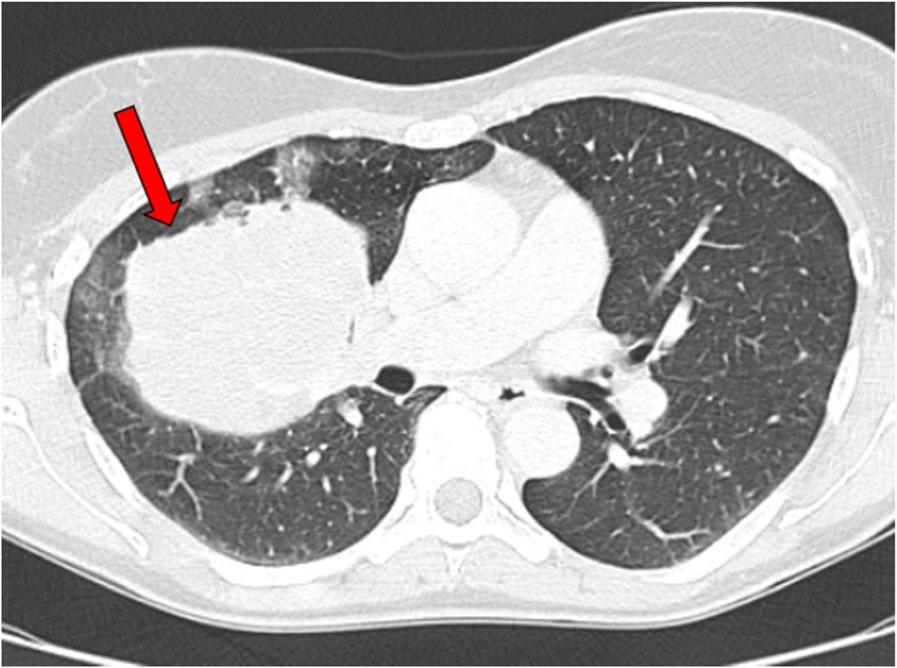
CT scan of chest performed in October 2014 because of the persistence of chronic cough, showing a mass of 90 mm in the right upper lobe (case report 1)

**Fig. 2:**
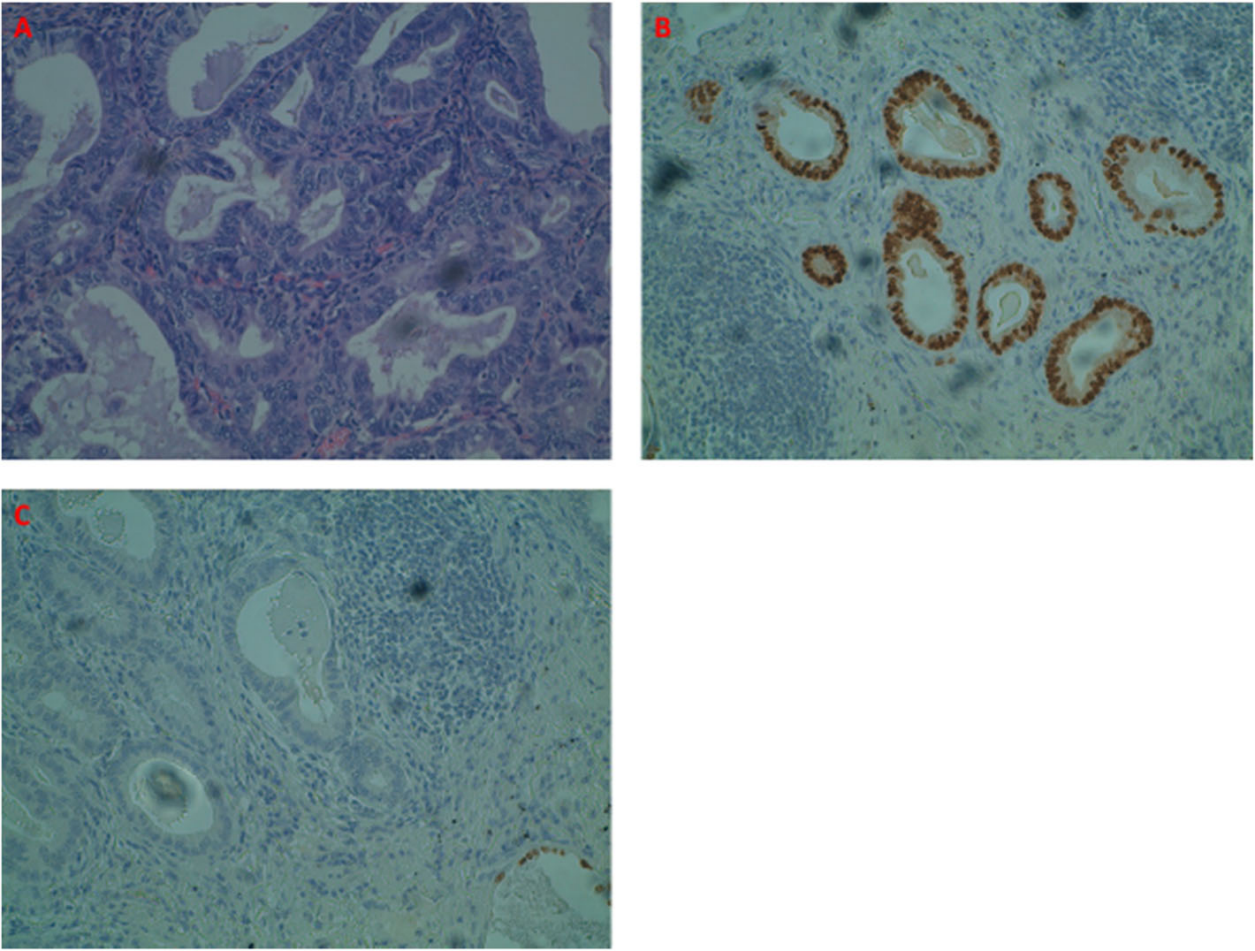
Histological examination of right lung lobectomy, showing tumoral cells colored with hematoxylin-eosin (**a**), positivity for estrogen receptor (ER) (**b**), and negativity for thyroid transcription factor 1 (TTF-1) (**c**)

A 56-year-old woman underwent ultrasonography of the abdomen in 1998 because of the appearance of abdominal swelling and pain. The exam revealed a pelvic mass, which was then confirmed to be uterine by an exploratory laparoscopy. Laparoscopy was therefore converted in laparotomy, and a hysterectomy with bilateral salpingo-oophorectomy without lymphadenectomy was performed. Histological examination of the surgical sample revealed a well-differentiated, estrogen receptor [ER] positive, endometrioid cancer with negative surgical margins. Preoperative staging performed with a total-body CT scan showed no distance metastasis, and FIGO stage was IB according to the 1996 edition [6]. Surgery was radical and, according to the guidelines at the time, the patient was considered low risk of relapse, then no adjuvant treatment was prescribed. Oncological follow-up was carried out with semestral clinical examination and CT scan, and it was negative during the following 5 years. In October 2014, the patient developed a chronic cough and underwent a CT scan of the chest, which revealed a 90 mm in diameter mass in the upper lobe of the right lung (Fig. 1). Full staging with total-body CT scan, gastroscopy, and colonoscopy did not show a primary tumor or other metastases. Bronchoscopy with biopsy revealed endometrial carcinoma cells staining positively for the ER. Immunostaining for Ki-67 was positive in 30% of malignant cells. Following multidisciplinary evaluation, the patient underwent neoadjuvant chemotherapy with carboplatin area under the curve (AUC) 5 plus paclitaxel 175 mg/m^2^ every 3 weeks. The first tumor imaging performed after three cycles of chemotherapy showed a partial response. Upon completion of 6 cycles of neoadjuvant chemotherapy, in February 2015, the patient underwent right lung lobectomy. Histopathology confirmed metastasis from endometrial carcinoma, which was ER positive, paired box 8 [PAX8] positive, thyroid transcription factor 1 [TTF-1] negative, and CDX2 negative (Fig. 2). Surgical margins were negative. After surgery, the patient was prescribed endocrine therapy with tamoxifen 20 mg/day. At the time of this writing, the patient is continuing hormonal treatment and oncological follow-up and remains disease free.

### Case report 2

**Fig. 3:**
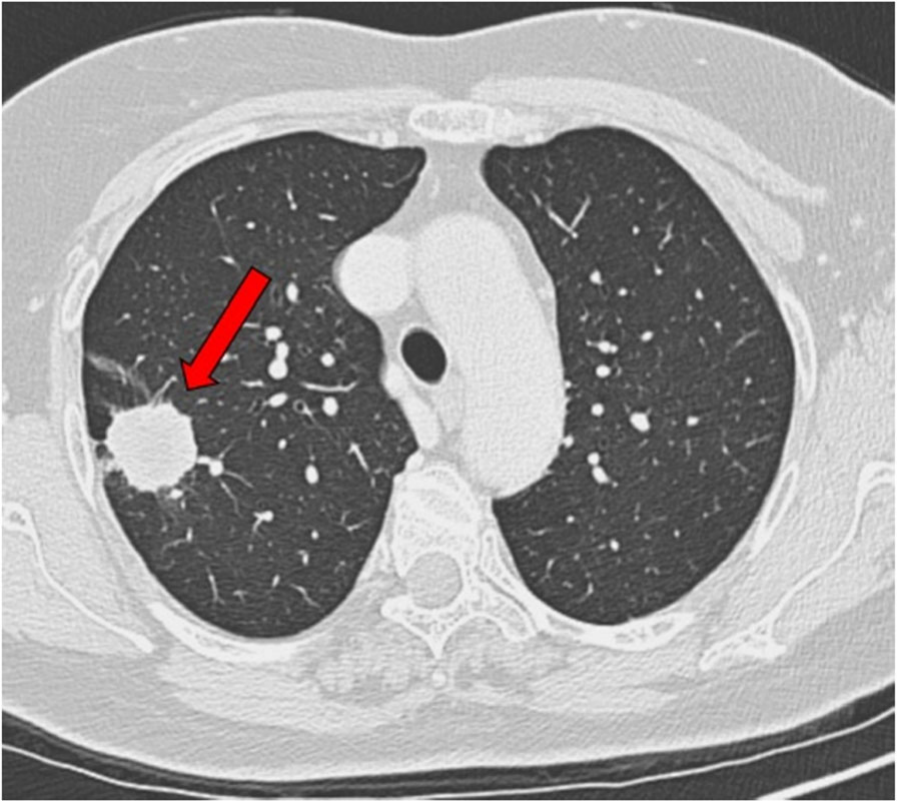
CT scan of chest performed in January 2019 to exclude pulmonary embolism, showing a mass of 28 mm in the right upper lobe (case report 2)

**Fig. 4:**
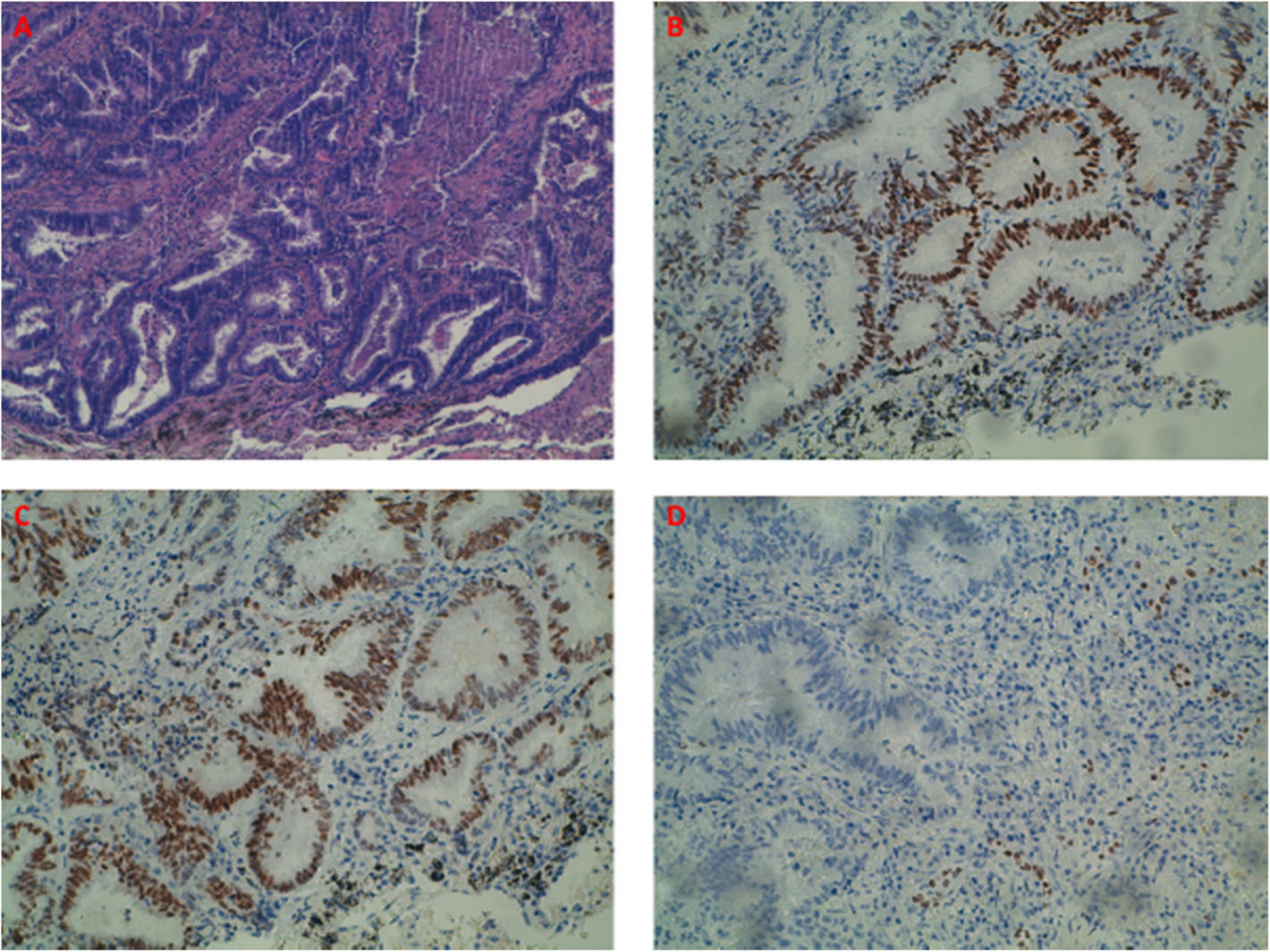
Histological examination of pulmonary metastasectomy, showing tumoral cells colored with hematoxylin-eosin (**a**), positivity for estrogen receptor (ER) (**b**), positivity for paired box 8 (PAX8) (**c**), and negativity for thyroid transcription factor 1 (TTF-1) (**d**)

A 75-year-old woman underwent hysterectomy with bilateral salpingo-oophorectomy and lymphadenectomy because of the accidental discovery of a pelvic mass in 1997. Histological examination of the surgical sample showed a grade 1 differentiated, ER positive, PAX8 positive, TTF-1 negative, and CA125 negative endometrioid carcinoma. Preoperative staging performed with total-body CT scan showed no distance metastasis, and FIGO stage was II according to the 1996 edition [6]. According to Italian guidelines at the time, the patient underwent adjuvant radiotherapy with 40 Gray delivered by external beams. The following 5 years of oncological follow-up, with semestral clinical examination and CT scan, were uneventful. In January 2019, the patient was admitted to the emergency department of our hospital because of worsening dyspnea. While excluding pulmonary embolism, a computed angiography of the chest showed a 28 mm in diameter mass in the upper lobe of the right lung (Fig. 3). A positron-emission tomography (PET) with fludeoxyglucose showed intense uptake of the tracer in the lung lesion (standardized uptake value [SUV] 12.2). Histopathology of a biopsy of the lesion revealed endometrial carcinoma cells, which were ER and PAX8 positive, and TTF1 and CA125 negative. Because of no other systemic metastases, in March 2019, the patient underwent surgical resection of the lesion. Histopathology confirmed the endometrial origin (ER and PAX8 positive, and TTF1, CDX2, and CA125 negative) and showed clear surgical margins (Fig. 4). After 4 months of follow-up, the patient developed a painful cutaneous node in the abdominal wall of the right hypochondrium. A total-body CT scan showed a 45 mm in diameter lung mass near the site of metastasectomy, a 33 mm in diameter node of the abdominal wall, and other two smaller lesions in the muscles of the abdominal wall. The cutaneous node was surgically removed, and the histological examination revealed metastasis from endometrial carcinoma, ER and PAX8 positive, and TTF1, CDX2, and CA125 negative. Vascular carcinoma microemboli were observed in the surrounding adipose tissue. Because of systemic disease, our multidisciplinary team decided for first-line chemotherapy, which consisted of carboplatin AUC5 and paclitaxel 175 mg/m^2^ every 21 days. At the time of this writing, therapy is still ongoing, 22 years after the first diagnosis of endometrial carcinoma.

## Brief review of literature

Systemic metastases from endometrial carcinoma usually develop within the first 3 years after radical treatment, while long-term recurrences are possible but uncommon [5]. Indeed, only a few cases of recurrences after more than 10 years after radical treatment are described in literature. The first experience was reported in 1987 by Lederman et al. A 47-year-old woman who received an intracavitary radium implant followed by radical hysterectomy for endometrial carcinoma. She developed a vaginal recurrence 26 years after local treatment. Treatment consisted of vaginal radiotherapy, after which the patient resumed follow-up with no further events [7]. In 2001, Ito et al. described a case of a 60-year old patient who underwent surgical resection of suspected lung cancer. However, histopathology revealed cells of adenoacanthoma, which were consistent with the diagnosis of endometrial adenoacanthoma dating back 17 years, when she had undergone radical hysterectomy. Staging workup showed no other sites of metastasis, and the patient resumed the oncological follow-up with no further events [8]. Lorenz et al. described a case of late recurrence of endometrial cancer in the abdominal scar of a patient who had undergone radical hysterectomy and radiotherapy for endometrial carcinoma 14 years before. The mass, which was 80 mm in diameter was surgically removed, and histopathology confirmed the diagnosis of metastasis from endometrial carcinoma [9]. Tsurumaki et al. described an unusual site of recurrence 11 years after radical hysterectomy. A 72-year-old woman underwent radical hysterectomy for endometrioid carcinoma and a lymph-node recurrence next to the left ureter 3 years after. This recurrence was treated with radical excision and radiotherapy. Eleven years after hysterectomy, the patient developed progressive left hydronephrosis. A CT scan of the abdomen showed a pelvic mass involving the left ureter. The patient underwent a left nephroureterectomy with partial cystectomy, and histopathology confirmed metastasis from endometrioid carcinoma. The patient resumed the follow-up after adjuvant radiotherapy [10]. In 2015, Yechieli et al. described a case of a 61-year-old female who underwent a total abdominal hysterectomy with bilateral salpingo-oophorectomy and lymph node for an endometrioid adenocarcinoma. Adjuvant external beam radiotherapy was performed, and the patient remained disease free for 17 years until an isolated vaginal cuff recurrence developed. The patient underwent salvage high-dose-rate brachytherapy, after which follow-up was resumed and continued until she died from cancer-unrelated causes [11]. Another unusual site of metastasis was reported by Addison et al., who described a case of a 62-year-old woman who had undergone radical hysterectomy for endometrial carcinoma in 2000. She needed emergency surgery in 2010 because of an acute abdomen and sepsis. Surgery consisted of laparotomy with appendicectomy. The histological examination confirmed appendicitis with peritonitis, but endometrial adenocarcinoma cells were also detected as an unexpected finding. After recovery, the patient was referred to an oncology team but received no further treatment [12]. Finally, in 2015, Franchello et al. reported the case with the most extended interval between surgery and relapse, 28 years. A 72-year-old woman with a history of multiple neoplasms, including endometrial carcinoma, underwent hysterectomy for endometrial carcinoma in 1989. A screening colonoscopy performed in 2013 showed a rectal mass. Histopathology of a biopsy of the rectal mass showed cancer cells of endometrial origin. The patient underwent surgical removal of the rectal mass and of a single liver metastasis discovered during the pre-surgical staging workup. Histopathology confirmed that both lesions were recurrences of endometrial carcinoma. No adjuvant treatments were indicated, and further follow-up was uneventful [13]. Table 1 summarizes the cases described in this paragraph.

**Table 1:** Cases of late recurrence of endometrial carcinoma reported in literature

Study	Year	Age at recurrence	Histology of primary tumor	Stage (FIGO)	Primary surgery	Site of recurrence	Time to recurrence	Treatment of recurrence
Lederman et al.	1987	47	Adenoacanthoma	IA	H	Vagina	26 years	Radiotherapy
Ito et al.	2001	60	Adenoacanthoma	IB	H	Lung	17 years	Surgery
Lorenz et al.	2004	73	Endometrial carcinoma	IA	H	Abdominal scar	14 years	Surgery
Tsurumaki et al.	2009	72	Endometrioid adenocarcinoma	II	H + L	Ureter	11 years	Surgery + radiotherapy
Yechieli et al.	2011	61	Endometrioid adenocarcinoma	IC	H + BSO + L	Vagina	17 years	Brachytherapy
Addison et al.	2012	62	Endometrial carcinoma	IB	H + BSO	Appendix	10 years	Surgery
Franchello et al.	2015	72	Endometrioid adenocarcinoma	IB	H + BSO + L	Rectum	28 years	Surgery
Case report 1		56	Endometrial carcinoma	IB	H + BSO	Lung	16 years	Surgery + CT + HT
Case report 2		75	Endometrial carcinoma	II	H + BSO + L	Lung and soft tissues	22 years	Surgery + CT

*H* hysterectomy, *BSO* bilateral salpingo-oophorectomy, *L* lymphadenectomy, *CT* chemotherapy, *HR* hormone therapy

## Discussion

The prognosis of endometrial carcinoma has constantly been improving over the last decades [14]. While excellent rates of survival can be achieved in patients with low-stage disease treated with local treatment alone, the addition of chemotherapy may reduce the risk of relapse in patients at higher risk. Local or distant recurrence, which usually occurs in the initial 3 years after local treatment, remains a problem for a proportion of patients [5]. Conversely, because the risk relapse beyond 5 years is low, guidelines limit oncology follow-up to the initial 5 years after local treatment [1]. In this paper, we describe two cases of patients who developed very late recurrences and were treated at our center. It is important to underline that these cancers were treated over 20 years ago according to guidelines that undergone several updates over the following years. In particular, the stratification of risk, critical as regards the decision about the adjuvant treatment, nowadays is no longer based only on FIGO staging and histological grade. In fact, different algorithms have been developed with the aim to create a risk stratification system based on several clinico-pathological parameters, which include not only FIGO stage and tumor differentiation grade but also age, histotype, depth of myometrial invasion, and lymphovascular space invasion [15]. Anyway, with the two cases described in this paper, the total of late relapses from endometrial carcinoma published in the medical literature adds up to nine. Due to such a low number, it is difficult to identify predictors of late recurrence, which can direct specific follow-up strategies. The median age was 62 years. Overall, seven out of nine patients (78%) were stage I FIGO, and the remaining two were stage II FIGO. Histologically, two tumors were defined as acanthomas, and the other seven are generically described as “endometrial carcinoma” or as “endometrioid adenocarcinoma,” the most frequent histological variant of endometrial carcinoma [16]. Primary local treatment consisted of hysterectomy in the four patients diagnosed before 2009, and hysterectomy plus bilateral salpingo-oophorectomy in the five patients diagnosed more recently. The recurrence time spanned from 10 to 28 years, with three patients recurring after more than 20 years. A common feature of these cases is that patients recurred with oligometastatic disease, and surgical removal or local treatment of recurrent disease was feasible in almost all cases, except the one described by Addison et al. For seven patients, further follow-up after local systemic treatment was uneventful. Only the two patients described in this paper received systemic therapy, one with neoadjuvant and the other with palliative intent. Currently, histopathology and molecular pathology play a critical role in identifying prognostic factors in several cancers. Even in endometrial cancer, the molecular pathology influences more and more the decision-making process about surgery, the need for adjuvant therapy, and follow-up management [17]. Unfortunately, the cases described here are few, and the information on further histopathological and molecular details are missing. Only the paper of Franchello et al. provides some information about the immunophenotypical pattern of the neoplasm: the rectal recurrence of endometrial carcinoma was positive for CK7, ER, antivimentin antibody, and PAX8, and negative for CK20 and CDX2. This profile allowed the differential diagnosis of primary colorectal cancer [18, 19]. The two cases that we describe in this manuscript display similar immunophenotypical features (ER and PAX8 positive and CDX2 negative). These immunohistochemical features usually identify low-grade, slowly growing endometrial cancers [20] and cannot be used to identify patients at risk for late relapse. Intriguingly, ER-positive breast cancer can recur systemically even after decades from surgery of primary tumor [21]. This phenomenon is due to “cell dormancy” and is less pronounced, if not absent, in other subtypes of breast cancer. ER expression and metabolism may influence the ability of endometrial cancer cells to migrate and to enter a dormant status similarly to breast cancer cells. Aging and related pro-tumorigenic stimuli as chronic inflammation may “awake” dormant cells and give rise to overt clinically overt metastases [22].

## Conclusion

Relapses of endometrial carcinoma rarely occur beyond 5 years from surgery. Yet, despite extremely rare, they are possible. While a change in the follow-up policies is not justified based on the very few case reports described here, it is crucial to consider endometrial cancer relapse in the differential diagnosis of metastases of unknown origin in women with previous adenocarcinoma of the endometrium, even if radical treatment has been performed many years before.

## Data Availability

Not applicable.

## Abbreviations

FIGO: Federation of Gynecology and Obstetrics
ER: Estrogen receptor
PAX8: Paired box 8
TTF-1: Thyroid transcription factor 1
SUV: Standardized uptake value
